# Injury characteristics and outcome of road traffic crash victims at Bugando Medical Centre in Northwestern Tanzania

**DOI:** 10.1186/1752-2897-6-1

**Published:** 2012-02-09

**Authors:** Phillipo L Chalya, Joseph B Mabula, Ramesh M Dass, Nkinda Mbelenge, Isdori H Ngayomela, Alphonce B Chandika, Japhet M Gilyoma

**Affiliations:** 1 Department of Surgery, Catholic University of Health and Allied Sciences-Bugando, Mwanza, Tanzania; 2 Department of Orthopaedic and Traumatology, Catholic University of Health and Allied Sciences-Bugando, Mwanza, Tanzania

**Keywords:** Road traffic crashes, Victims, Injury characteristics, Outcome, Tanzania

## Abstract

**Background:**

Road traffic crash is of growing public health importance worldwide contributing significantly to the global disease burden. There is paucity of published data on road traffic crashes in our local environment. This study was carried out to describe the injury characteristics and outcome of road traffic crash victims in our local setting and provide baseline data for establishment of prevention strategies as well as treatment protocols.

**Methods:**

This was a prospective hospital based study of road traffic crash victims carried out at Bugando Medical Centre in Northwestern Tanzania between March 2010 and February 2011. After informed consent to participate in the study, all patients were consecutively enrolled into the study. Data were collected using a pre-tested questionnaire and analyzed using SPSS computer software version 15.0.

**Results:**

A total of 1678 road traffic crash victims were studied. Their male to female ratio was of 2.1:1. The patients ages ranged from 3 to 78 years with the mean and median of 29.45 (± 24.22) and 26.12 years respectively. The modal age group was 21-30 years, accounting for 52.1% patients. Students (58.8%) and businessmen (35.9%) were the majority of road traffic crash victims. Motorcycle (58.8%) was responsible for the majority of road traffic crashes. Musculoskeletal (60.5%) and the head (52.1%) were the most common body region injured. Open wounds (65.9%) and fractures (26.3%) were the most common type of injuries sustained. The majority of patients (80.3%) were treated surgically. Wound debridement was the most common procedure performed in 81.2% of the patients. The complication rate was 23.7%. The overall average length of hospital stay (LOS) was 23.5 ± 12.3 days. Mortality rate was 17.5%. According to multivariate logistic regression analysis, patients who had severe trauma (Kampala Trauma Score II ≤ 6) and those with long bone fractures stayed longer in the hospital and this was significant (P < 0.001) whereas the age of the patient, severe trauma (Kampala Trauma Score II ≤ 6), admission Systolic Blood Pressure < 90 mmHg and severe head injury (Glasgow Coma Score = 3-8) significantly influenced mortality (P < 0.001).

**Conclusion:**

Road traffic crashes constitute a major public health problem in our setting and contribute significantly to unacceptably high morbidity and mortality. Urgent preventive measures targeting at reducing the occurrence of road traffic crashes is necessary to reduce the morbidity and mortality resulting from these injuries. Early recognition and prompt treatment of road traffic injuries is essential for optimal patient outcome.

## Background

Road traffic crashes (RTCs) are a major cause of misery, disability and death globally, with a disproportionate number occurring in developing countries [1-3]. The problem is increasing at a fast rate in developing countries due to rapid motorization and other factors [4]. In addition, while low-income and middle-income countries already account for more than 85% of all road traffic deaths in the world, the upsurge in the number of vehicles per inhabitant will result in an anticipated 80% increase in injury mortality rates between 2000 and 2020 [5]. It has been predicted that by 2020, road traffic injuries will rank as high as third among causes of disability adjusted life years (DALYs) lost [2,5,6].

Tanzania, a developing country in Africa, has witnessed at least a fivefold rise in recorded traffic-related fatalities during the last decade [7]. This in part is due to the proliferation of roads, which are often in poor states and also, a phenomenal increase in the number of motor vehicles, many of which are old, and not road-worthy. The increasing use of motorcycles particularly for commercial service is a source of concern in this regard because motorcycles cause many more fatal road crashes than other vehicles worldwide. As motorcycles are relatively unsafe vehicles, the riders must be considered as unprotected vehicle users and their injuries are usually severe [8].

The injury characteristics for road traffic crashes in developing countries differs in important ways from the profile seen in developed countries, and it can provide guidance for making policies to improve prevention and control. Pedestrians are most vulnerable to injury and death. This may be due to a number of factors, including lack of pedestrian facilities in road design, poor knowledge and practice of road safety measures by the general population, recklessness behaviour of motorists, high speed driving, and low levels of vehicle ownership. The high proportions of passenger fatalities appear to be associated with extensive use of public transport, types and condition of such vehicles and the driving skill of their operators [2,9]. Addressing the risks of these groups will require multiple policy initiatives. To be effective, policies on traffic safety in developing countries must be based on local evidence and research, and designed for the particular group and economic circumstances found in developing countries [10].

In Sub-Saharan Africa including Tanzania, with increasing vehicular traffic, RTI has become an immense health problem and constitutes a burden on under-funded and oversubscribed health services [11,12]. The reasons for the high burden of road traffic crashes in developing countries are: growth in the numbers of motor vehicles; higher number of people killed or injured per crash in low-income countries, poor enforcement of traffic safety regulations; inadequacy of health infrastructure, and poor access to health care [2,7,11,12].

Injuries related to road traffic crashes contribute significantly to the number of trauma admissions at Bugando Medical Centre, taking out a significant number of lives and resources including consumables and the health worker time. However, despite this burden, there is little, if any, published information on RTCs in our local environment and the public policy responses to this problem have been muted, probably because of lack of local data regarding the problem. This study was conducted in order to provide baseline data to policy makers and other stakeholders who may wish to undertake interventions to improve road safety in the country.

The aim of this study therefore, was to describe the injury characteristics and outcome of road traffic crashes in our setting. The study provides basis for establishment of prevention strategies as well as treatment protocols.

## Methods

### Study setting and design

This was a prospective hospital based study of road traffic crash patients of all age groups and gender presenting to the Accident and Emergency (A&E) department of Bugando Medical Centre (BMC) between March 2010 and February 2011. Bugando Medical Centre (latitude 2.52680 S; longitude 32.9062 E) is a 1000-bed, tertiary care and teaching hospital for the Catholic University of Health and Allied Sciences-Bugando, Mwanza, Tanzania (CUHAS). It is located in Mwanza city, along the shores of Lake Victoria in north-western Tanzania. Tanzania is located in East Africa between longitude, 29° and 41° east and latitude 1° and 12° south. Tanzania borders Kenya to the north, Rwanda, Burundi, and the Democratic Republic of Congo to the west, and Zambia, Malawi and Mozambique to the south. The total population in Tanzania was last reported at 43.2 million people in 2010. The hospital provides service to a population of approximately13 million people from its neighboring six regions in northwestern Tanzania (Mwanza, Mara, Kagera, Shinyanga, Kigoma and Tabora). There is no trauma centre or established advanced pre-hospital care in Mwanza city as a result all trauma patients are referred to BMC for expertise management.

### Study subjects

Subjects for the study included all road traffic crash victims of all age groups and gender irrespective of injury severity who was managed at BMC during the study period and who consented for the study. Patients who failed to give proper information and those who had no relative to consent for the study were excluded from the study. Recruitment of patients to participate in the study was done at the A & E department. Patients were screened for inclusion criteria and those who met the inclusion criteria were, after informed consent to participate in the study, consecutively enrolled into the study.

All study patients were first resuscitated in the A & E department according to Advanced Trauma Life Support. From the A & E department patients were taken into the surgical wards or the intensive care unit (ICU) from where necessary investigations were completed and further treatment was instituted. Variables studied included demographic profile (age, sex, and occupation), mechanism of injury, prehospital care, contributing factors to RTC, injury arrival interval, trauma scores, body region injured, treatment offered, complications of treatment. Outcome variables were length of hospital stay, mortality and disability. The severity of injury was determined using the Kampala trauma score II (KTS II) [13]. Severe injury consisted of a KTS II ≤ 6, moderate injury 7-8, and mild injury 9-10. Patients with head injuries were classified according to Glasgow Coma Scale (GCS) into: severe (GCS 3-8), moderate (GCS 9-12) and mild (GCS 13-15). Depending on the type of injury, the patients were treated either conservatively or by surgery. All patients were followed up till discharged or death. This information was collected using a pre-tested questionnaire.

### Statistical data analysis

Statistical data analysis was done using SPSS software version 15.0. Data was summarized in form of proportions and frequent tables for categorical variables. Continuous variables were summarized using means, median, mode and standard deviation. P-values were computed for categorical variables using

Chi-square (χ^2^) test and Fisher's exact test depending on the size of the data set. Independent student t-test was used for continuous variables. Multivariate logistic regression analysis was used to determine predictor variables that are associated with outcome. A p-value of less than 0.05 was considered to constitute a statistically significant difference.

### Ethical considerations

The study was carried out after the approval by the department of surgery and BMC/CUHAS-Bugando ethics review board. An informed written consent was sought from patients or relatives.

## Results

### Demographic profile

During the period under study, a total of 1678 road traffic crash victims (1624 road traffic crashes) were studied. Males were 1128 (67.2%) and females were 550 (32.8%), giving a male to female ratio of 2.1:1. The patients ages ranged from 3 to 78 years with the mean of 29.45 ± 24.22 years. The median and the mode were 26.12 and 24.00 years respectively. The modal age group was 21-30 years, accounting for 874 (52.1%) patients. The vast majority of patients, 998 (59.5%) were self-employed and most of them, 972 (57.9%) had either primary or no formal education. Students and businessmen were the majority of road traffic crash victims accounting for 987 (58.8%) and 603 (35.9%) of cases respectively. This was followed by public servants 36 (2.1%), peasants 22 (1.3%) and preschool children 20 (1.2%).

### Circumstances of the injury

Motorcycle (986, 58.8%) was responsible for the majority of road traffic crashes, followed by motor-vehicles (650, 38.7%), bicycle (36, 2.1%) and other means of transport (e.g. donkey, trolley etc) in 4 (0.2%) of cases. Pedestrians (930, 55.4%) accounted for the majority of victims, followed by passengers (457, 27.2%), drivers/riders (287, 17.2%) and others (4, 0.2%).

Regarding the time of the crash, 1016 (60.5%) crashes occurred during the day, 584(34.8%) at night. In 78 (4.6%) crashes, the time was not specified. Helmet and seat belt use among motorcyclists and occupants of vehicles were recorded in 24.7% (i.e. 244 out of 986) and 13.5% (i.e. 88 out of 650) of patients respectively.

History of alcohol consumption prior to the accident was reported in 289 (17.2%) patients.

The vast majority of patients (1109, 66.1%) reported to the A & E department within 24 hours after injury. None of the patients received any pre-hospital care and majority of them (1284, 76.5%) were brought in by relatives, friends or Good Samaritan, 380 (22.6%) by police and only 14 (0.8%) patients were brought in by ambulance. The waiting time, defined as the time interval taken from reception at the A & E department and reception of treatment ranged from 15 minutes to 8 hours with the mean (± standard deviation) and median of 2.24 ± 1.26 hours and 2.00 hours respectively. The majority of patients (1120, 66.7%) were attended to within 4 hours of arrival to the A & E department.

### Injury characteristics

Musculoskeletal (extremities) and the head were the most common body region injured accounting for 60.5% and 52.1% of cases respectively (Table 1). Musculoskeletal injuries commonly affected the lower limbs (785, 77.3%). Isolated injuries occurred in 1289 (76.8%) patients while 389 (23.2%) patients had multiple injuries. Open wounds (i.e. bruises, abrasions, lacerations, crush wounds, traumatic amputation, etc) and fractures were the most common type of injuries sustained (Table 2).

**Table 1:** Site of injuries among the victims

Site of injury	Frequency	Percentage
Musculoskeletal (extremities)	1015	60.5
Head	874	52.1
Abdomen	808	48.3
Chest	741	44.2
Maxillofacial	123	7.3
Pelvis	35	2.1
Spines	12	0.7

**Table 2:** Type of injuries among the victims

Type of injury	Frequency of injuries	Percentage
**Open wounds**	**1106**	**65.9**
**Fractures**	**442**	**26.3**
Lower limb fractures	182	41.2
Upper limb fractures	132	29.9
Skull/maxillofacial fractures	87	19.7
Pelvic fractures	16	3.6
Rib fractures	14	3.2
Spinal fractures	6	1.4
Clavicle fractures	5	1.1
**Intracranial hemorrhages**	**282**	**16.8**
Subdural	82	29.1
Epidural	74	26.2
Subarachnoid	64	22.7
Intracerebral	62	22.0
**Visceral injuries**	**124**	**7.4**
Spleen	58	46.8
Intestines	24	19.4
Liver	20	16.1
Urinary bladder	14	11.3
Kidney	10	8.1
Other visceral injuries **Traumatic limb amputations** **Pneumothorax** **Hemothorax** **Other injuries**	8 **40** **16** **7** **12**	6.5 **23.8** **1.0** **0.4** **0.7**

According to Kampala Trauma Score II (KTS II) (Table 3), the majority of patients sustained moderate injuries (KTS II = 7-8) in 942 (56.1%). Severe injuries (KTS II ≤ 6) and mild injuries (KTS II = 9-10) were recorded in 648 (38.6%) and 88 (5.3%) patients respectively.

**Table 3:** Kampala Trauma Score (KTS II) Description

	Description	Score
**A**	**Age (in years)**	
	5-55	1
	< 5 or > 55	0
**B**	**Systolic Blood Pressure on admission**	
	More than 89 mm Hg	2
	Between 89-50 mm Hg	1
	Equal or below 49 mm Hg	0
**C**	**Respiratory rate on admission**	
	0-29/minute	2
	30+	1
	< or = 9/minutes	0
**D**	**Neurological status**	
	Alert	3
	Responds to verbal stimuli	2
	Responds to painful stimuli	1
	Unresponsive	0
**E**	**Score for serious injuries**	
	None	2
	One injury	1
	More than one	0

Kampala Trauma Score total = A+B+C+D+E

The mortality rates in patients with mild, moderate and severe injuries were 6.8% (12 deaths), 23.9% (42 deaths) and 69.3% (122 deaths) respectively. According to multivariate logistic regression analysis, these differences were statistically significant (P < 0.001). The Glasgow coma scale indicated that most of the patients (487, 55.7%) sustained mild head injury, 302 (34.6%) patients sustained moderate head injury and 85 (9.7%) patients had severe head injury. Patients with severe head injuries had significant high mortality rates (64.2%, 113 deaths) compared with patients who had moderate (33.0%, 58 deaths) and mild (2.8%, 5 deaths) head injuries (P < 0.001).

### Admission pattern and treatment

The majority of patients, 1018(60.7%) were admitted in the general surgical wards. 412 (24.6%) patients had an overnight stay at the Accident & Emergency department and then discharged. 248 (14.8%) patients were admitted in the intensive care unit (ICU); of these, 159 (64.1%) necessitated ventilatory support. The majority of patients, 1348 (80.3%) were treated surgically. Wound debridement was the most common procedure performed in 81.2% of patients (Table 4).

**Table 4:** Type of surgical procedures performed (N = 1348)

Type of surgical procedure	Frequency	Percentage
Wound debridement	1095	81.2
Treatment of fractures	400	29.7
Craniotomy/burr holes	208	15.4
Exploratory laparotomy Limb re-amputation Skin grafting	112 34 22	8.3 25.2 16.3
Underwater seal drainage (UWSD)	20	1.5
Other surgical procedures	8	0.6

### Complications among the victims

A total of 484 complications were recorded in 398 patients giving a complication rate of 23.7%. Of these, wound sepsis was the most common complication accounting for 79.3% 0 f cases (Table 5).

**Table 5:** Complications among the victims

Complications	Frequency	Percentage
Wound sepsis	384	79.3
Complications of fractures	98	20.2
Complications of abdominal surgery	34	7.0
Hemorrhagic shock	26	5.4
Tetanus	10	2.1
Skin grafting failure	6	1.2
Subcutaneous emphysema	3	0.6
Empyema thoracis	2	0.4
Re-amputation	2	0.4
Neurological deficit	1	0.2

### Clinical outcome of road traffic crash victims

**Figure 1:**
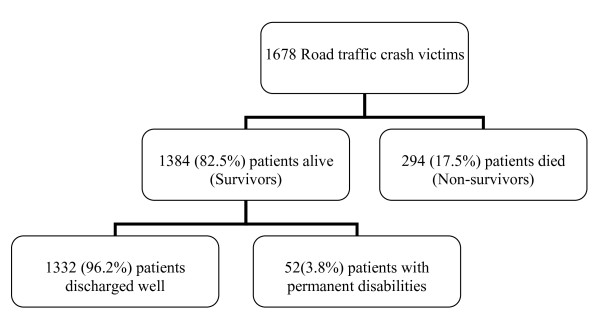
**Flow chart showing the clinical outcome of road traffic crash victims**.

Of the 1678 patients, 1384 (82.5%) patients were alive. Of these, 1332(96.2%) patients were discharged well without permanent disability and the remaining 52(3.8%) patients were discharged with permanent disabilities such as limb amputations in 40 (23.8%) patients, permanent neurological deficit in 5 patients, severe spinal injuries with paraplegia in 4 patients, post-traumatic seizures in 2 patients and traumatic penile amputation in 1 patents. There were 294 deaths, accounting for an overall mortality rate of 17.5% (Figure 1).

The overall length of hospital stay (LOS) ranged from 1 day to 136 days with the mean of 23.5 ± 12.3 days. The median was 22.4 days. LOS for non-survivors ranged from 1 day to 34 days (mean 6.7 ± 1.4 days and median 5.6 days). The length of ICU stay ranged from 1 day to 38 days (mean = 8.9 ± 2.4 days, median 6.3 days). According to multivariate logistic regression analysis, patients who had severe trauma (Kampala Trauma Score II ≤ 6) and those with long bone fractures stayed longer in the hospital and this was significant (P < 0.001) whereas the age of the patient, severe trauma (Kampala Trauma Score II ≤ 6), admission Systolic Blood Pressure < 90 mmHg and severe head injury (Glasgow Coma Score = 3-8) significantly influenced mortality (P < 0.001).

## Discussion

In this review, the majority of road traffic crash victims were young in their most reproductive and productive years and showed a male preponderance. The young male preponderance in the present study agrees with findings reported elsewhere [8,14-17]. This group represents the economically active age and portrays an economic lost both to the family and the nation and the reason for their high incidence of road traffic crash reflects their high activity levels and participation in high-risk activities such as recklessness driving/riding, over-speeding, driving/riding under the influence of alcohol and driving/riding without wearing any protective gears. Male predominance in this study is due to their increased participation in high-risk activities. The fact that the economically productive age-group were mostly involved demands an urgent public policy response.

Students and businessmen were the most injured because of the rush through heavy traffic to get to their businesses and to the school. Similar observation was noted in the previous study by others [8,14]. Students are usually involved in road traffic crashes as they rush through heavy traffic to and from their schools. These school-age group children are usually very active and are often less supervised than pre-school age children. Coupled with the paucity of boarding school facilities for children of their age as well as of school buses, schoolchildren have to walk varying distances to and from school. This was the case in the vast majority of children knocked down in this study. As students formed one of the largest groups of road traffic victims, an improved school transportation system may reduce the incidence of road traffic crashes. Businessmen are often involved in buying and selling which necessitates movement from one place to another. This often involves travelling with good purchased, and in order to maximize profits, they usually opt for the cheapest means of transport available such as motorcycles.

In this study, motorcycle was responsible for the majority of road traffic crashes accounting for 58.8% of cases. The prevalence of motorcycle injuries in this study is higher than that reported a year earlier by Chalya *et al *[8] reflecting increase in the magnitude of the problem in our setting. Motorcycle use is becoming popular in Tanzania as it has become a cheaper and easier means of transportation in most cities. However their use is characterized by non-helmet use by riders and their passengers, passenger overload, lack of certified driver training and valid licensing, over speed and reckless driving, poor regulation and law enforcement and possible use of alcohol and drugs [7,8]. In the present study 17.2% road traffic crash victims were found to have consumed alcohol prior to the accident. This is a higher proportion than 8%, 14.9% and 16.9% reported respectively by others [18-20]. The role of alcohol in impairing driving ability is well documented. Alcohol usage causes carelessness and loss of concentration as well as over speeding and neglecting to use safety equipment such as helmet.

In agreement with other studies [14,20,21], pedestrians (55.4%) accounted for the majority of road traffic victims in our study, but in contrast with other studies which reported passengers as the majority of cases [7,8]. High incidence of pedestrians in this study reflecting low public awareness on road use and therefore pedestrians are less likely to use walking pavements even if they are available. In addition, the absence of pedestrian walkways in most of the roads in developing countries like Tanzania has increased the vulnerability of pedestrians to all motorized vehicles [7,22].

The finding that most of injuries in the present study occurred during the day agrees with that of other studies [8,14,23]. Increased rate of injuries during the day can be explained by increased traffic jams as well as increased human activities in the city during the day time. Knowing the time of injury in trauma patient is important for prevention strategies.

In this study, helmet and seat belt use among motorcyclists and occupants of vehicles were recorded in 24.7% and 13.5% of patients respectively. The same trend of non-usage of crash helmet and seat-belts was demonstrated in other studies [8]. The low incidence of helmet and seat belt use among motorcyclist and occupants of vehicles in this study reflects increased risks of severe trauma and head injuries in this region. This observation calls for preventive measures focusing on safety belt and crash helmet use.

The prehospital care of trauma patient has been reported to be the most important factor in determining the ultimate outcome after the injury [8]. None of our patients had pre-hospital care; as a result the majority of them were brought in by relatives, Good Samaritan and police who are not trained on how to take care of these patients during transportation. Only 0.8% of cases were brought in by ambulance. Similar observation was reported by other studies [8,21,24]. This observation is common to many other developing countries.

Pre-hospital care is unsatisfactory in many countries, especially in low-and middle-income countries (LMICs) [21,24], where the majority of trauma deaths occur in the pre-hospital phase. In most LMICs, transport of road traffic victims, is usually provided by relatives, taxi drivers, truck drivers, police officers and other motorists; who are usually untrained [24]. The lack of advanced pre-hospital care and ineffective ambulance system for transportation of patients to hospitals are a major challenges in providing care for trauma patients in our environment and have contributed significantly to poor outcome of these patients. In most of developing countries like Tanzania, more than 80% of victims of road traffic crash have no definable source of private or governmental health care insurance at the time of their injury. Rapid arrival of the emergency medical services (EMS) at the crash scene and proper victim transportation by trained personnel may reduce injury severity and reduce the number of preventable deaths.

In agreement with previous studies [8,14,15,17], the present study found that musculoskeletal and head injuries were the most common body region injured attributing the latter to the low use of motorcycle helmets in our study; a situation seen in other developing countries. In the present study, no patient who was helmeted at the time of injury sustained head injury reflecting its importance in prevention of head injuries among motorcycle injury patients. Our high figure of musculoskeletal injuries affecting mainly the lower limbs is attributable to the large number of pedestrians. Pedestrians are unprotected road users and therefore they are highly exposed to high risk of limb injuries [25].

A number of scoring systems have been developed to facilitate consistent trauma triage, severity evaluation, management and prognostication [26]. These include the Injury Severity Score (ISS), Pediatric Trauma Score (PTS), Abbreviated Injury Score (AIS), Revised Trauma Score (RTS) and Trauma score and injury severity score (TRISS) [27]. In the present study, the severity of injury was determined using the Kampala trauma score II (KTS II) whose validity and reliability for use in both adults and children was described elsewhere [13]. This scoring system, compares favorably with other trauma scoring systems such as the Revised Trauma Score (RTS) and Injury Severity Score (ISS) [27]. It is scored based on age, number of serious injuries, systolic blood pressure, respiratory rate and neurologic status on presentation.

Initially, it was scored on a scale of 5-16. A severe injury consisted of a KTS less than 11, a moderate injury 11-13 and a mild injury 14-16. The KTS was then modified in 2004 to a range of 0-10. Although the parameters were maintained, the scoring of all the parameters except for blood pressure were given 1 score lower. Thus, mild injuries have a KTS of 9-10, moderate 7-8 and severe 6 or less [28].

Most of our patients were treated surgically, which is in agreement with other similar studies [8,14,15]. The high incidence of surgical treatment in our study is attributable to the high incidence of patients with moderate to severe injuries the majority of which required surgical intervention.

The current study had a mortality rate of 17.5%, which is higher than that reported in Rwanda by Twagirayezu *et al *[17]. High mortality rate in the present study was recorded in patients with severe trauma, admission SBP < 90 mmHg, severe head injury and in patients in their extremes of age.

The length of hospital stay (LOS) has been reported to be an important measure of morbidity among trauma patients. Prolonged hospitalization is associated with an unacceptable burden on resources for health and undermines the productive capacity of the population through time lost during hospitalization and disability. Our figures for the overall mean LOS in the present study were higher than that reported by others [8,17,29]. Prolonged LOS in our study is attributable to presence of major trauma patients and large number of patients with long bone fractures which took time to heal as majority of them were treated with either skeletal or skin traction and only few patients were treated with open reduction and internal fixation.

## Conclusion

Road traffic crashes constitute a major public health problem in our setting and the young adult male in their economically productive age-group are mostly involved. Students and businessman are the largest groups of road traffic crash victims. Limb and head injuries are the most common types of injury sustained predisposing these patients to prolonged hospitalization and mortality. Since the majority of road traffic crashes are preventable, enforcement of safety rules will help in reducing the occurrence of RTCs. Awareness campaigns concerning safety rules targeted at the high risk groups (young adult male, students and businessman) will also be of help in reducing the occurrence of road traffic crashes as well as improvement of the roads. Early recognition and prompt treatment of road traffic injuries is essential for optimal patient outcome.

## Competing interests

The authors declare that they have no competing interests.

## Authors' contributions

PLC--Study design, data analysis, manuscript writing & editing and submission of manuscript, JBM, DMR, NM, IHN and ABC participated in data analysis, manuscript writing & editing and JMG supervised the study and participated in designing the study, data analysis, manuscript writing & final editing of the study. All the authors read and approved the final manuscript.
